# Supplementary material for: AffyMAPSDetector: a software tool to characterize Affymetrix GeneChip™ expression arrays with respect to SNPs
Source: BMC Bioinformatics. 2007 Jul 30;8:276. doi: 10.1186/1471-2105-8-276 (PMC1959249; doi:10.1186/1471-2105-8-276)
Supplement: Additional file 13 — AffyMAPSDetector v1 source code. AffyMAPSDetector source code. [file 1471-2105-8-276-S13.zip › AffyMAPSDetector_v1_src/doc/affychip/snpdetector/ui/package-summary.html]

affychip.snpdetector.ui


|  |  |  |  |
| --- | --- | --- | --- |
| **Overview** \| **Package** \| Class \| **Use** \| **Tree** \| **Deprecated** \| **Index** \| **Help** | | |  |
| **PREV PACKAGE**   **NEXT PACKAGE** | **FRAMES**    **NO FRAMES**     **All Classes** |  |  |


---

## Package affychip.snpdetector.ui

|  |  |
| --- | --- |
| **Class Summary** | |
| **AffyMapsDetectorFrame** | Title: Probe SNP Detector |
| **AffyMapsDetectorUI** | Title: Probe SNP Detector |
| **CrossHbCheckFrame** | Title: Probe SNP Detector |
| **FileLoaderWizardDialog** | Title: Probe SNP Detector |
| **StepOnePanel** | Title: Probe SNP Detector |
| **StepThreePanel** | Title: Probe SNP Detector |
| **StepTwoPanel** | Title: Probe SNP Detector |

---


|  |  |  |  |
| --- | --- | --- | --- |
| **Overview** \| **Package** \| Class \| **Use** \| **Tree** \| **Deprecated** \| **Index** \| **Help** | | |  |
| **PREV PACKAGE**   **NEXT PACKAGE** | **FRAMES**    **NO FRAMES**     **All Classes** |  |  |


---
